# A preliminary study on predictors of treatment response to repetitive transcranial magnetic stimulation in patients with treatment‐resistant depression in Japan

**DOI:** 10.1002/npr2.12290

**Published:** 2022-08-30

**Authors:** Haruki Ikawa, Mamoru Tochigi, Yoshihiro Noda, Hiroshi Oba, Tatsuro Kaminaga, Keita Sakurai, Emi Ikebuchi, Naoki Hayashi, Hiroshi Kunugi

**Affiliations:** ^1^ Department of Neuropsychiatry Teikyo University School of Medicine, Graduate School of Medicine Itabashi Japan; ^2^ Tokyo‐Yokohama TMS clinic Kawasaki Japan; ^3^ Health Care Center, the University of Electro‐Communications Tokyo Japan; ^4^ Department of Neuropsychiatry Keio University School of Medicine Tokyo Japan; ^5^ Department of Radiology Teikyo University School of Medicine, Graduate School of Medicine Itabashi Japan; ^6^ Department of Radiology National Center for Geriatrics and Gerontology Obu Japan; ^7^ Teikyo Heisei University, Graduate School of Clinical Psychology Toshima Japan; ^8^ Nishigahara Hospital Kita Japan

**Keywords:** arterial spin labeling, dorsolateral prefrontal cortex, repetitive transcranial magnetic stimulation, treatment‐resistant depression, ventrolateral prefrontal cortex

## Abstract

**Background:**

Brain imaging studies have reported that the effect of repetitive transcranial magnetic stimulation (rTMS) is associated with the activities of the dorsolateral prefrontal cortex (DLPFC) and ventral medial prefrontal cortex (VMPFC). However, few studies have been conducted in Japanese patients.

**Aim:**

We aimed to identify brain regions associated with depressive symptom changes by measuring regional cerebral blood flow (rCBF) in the DLPFC and VMPFC before and after the high‐frequency rTMS to the left DLPFC in Japanese patients with treatment‐resistant depression.

**Method:**

Fourteen patients participated in the rTMS study and were assessed with the 17‐item Hamilton depression rating scale (HAM‐D_17_). Among them, 13 participants underwent magnetic resonance imaging scan of the brain using the arterial spin labeling method. The rCBF was calculated using the fine stereotactic region of interest template (FineSRT) program for automated analysis. We focused on eight regions reported in previous studies.

**Results:**

Depression severity significantly decreased after 2 week (HAM‐D_17_:11.4 ± 2.8, *P* = 0.00027) and 4 week (HAM‐D_17_: 11.0 ± 3.7, *P* = 0.0023) of rTMS treatment. There was no significant change in rCBF at each region in the pre‐post design. However, there was a significantly negative correlation between baseline rCBF in the right DLPFC and the improvement in HAM‐D_17_ score (*r* = −0.559, *P* = 0.047).

**Conclusion:**

We obtained supportive evidence for the effectiveness of rTMS to the prefrontal cortex in treatment‐resistant depression, which may be associated with reduced rCBF of the right DLPFC before initiation of rTMS.

## 1 INTRODUCTION

Repetitive transcranial magnetic stimulation (rTMS) is a therapeutic intervention method in which regular magnetic stimulation is continuously administered to the targeted cerebral cortex to neuromodulate the stimulation site and its related networks beyond the stimulation period.^1^, ^2^ In particular, rTMS is currently applied to the prefrontal cortex to improve depressive symptoms in patients with treatment‐resistant depression.^3^, ^4^ TMS was originally used as a neurophysiological examination tool. In the case of rTMS, high‐frequency stimulation (typically 10 Hz) is known to enhance excitability in the targeted cerebral cortex, while low‐frequency stimulation (<1 Hz) suppresses its function.^5^, ^6^ rTMS has advantages over existing treatments such as antidepressants and electroconvulsive therapy in terms of fewer adverse effects and noninvasiveness.^7^, ^8^

One of the main therapeutic targets of rTMS for depression is the dorsolateral prefrontal cortex (DLPFC), which is associated with cognitive functions such as working memory and executive function. In fact, positron emission tomography (PET) studies in depressed patients have reported decreased regional cerebral blood flow (rCBF) in this region.^9^, ^10^, ^11^ Standard rTMS treatment for depression involves high‐frequency stimulation of the left DLPFC, which increases cortical excitability at the stimulation site, indirectly suppressing hyperexcitability in the ventral medial prefrontal cortex (VMPFC), including the anterior cingulate cortex and subcallosal area, thereby improving depressive symptoms.^12^, ^13^ In particular, it has been repeatedly reported that increased rCBF in the anterior cingulate cortex prior to treatment can be a predictor of therapeutic response to rTMS.^13^, ^14^, ^15^, ^16^ Based on these findings, it is highly plausible that the DLPFC and VMPFC are involved in the depression‐related therapeutic mechanism of rTMS. In addition, previous studies using functional magnetic resonance imaging (fMRI) and electroencephalography (EEG) have suggested that the right prefrontal cortex may be hyperexcited relative to the left prefrontal cortex in the pathophysiology of depression.^17^ In fact, low‐frequency rTMS to the right DLPFC is known to exert an antidepressant effect by suppressing the hyperactivity of the right DLPFC.^18^

To date, most of the aforementioned studies were conducted in North America, Europe, and Australia, while few studies have been conducted in the Japanese population.^3^, ^16^, ^19^ Furthermore, pharmacotherapy algorithms and medical systems for treating depression differ between Japan and other countries. Therefore, reconfirming the treatment outcomes and side effects of rTMS treatment in Japan is important for developing the clinical application of rTMS in the future.

In this study, we aimed to identify brain regions associated with depressive symptom changes by measuring rCBF changes in the DLPFC and VMPFC regions before and after an acute course of rTMS in Japanese patients with treatment‐resistant depression.

## 2 METHOD

### 2.1 Participants

Participants were 14 patients who received rTMS treatment at the Department of Neuropsychiatry, Teikyo University Hospital, between July 2013 and February 2019. The participant's characteristics are as follows: eight males and six females; aged 39.8 ± 13.8 years; baseline score on the 17‐item Hamilton Depression Rating Scale (HAM‐D_17_)^20^: 17.9 ± 3.6; all right‐handed; three outpatients; and 11 inpatients (these values are presented as 18.7 ± 3.6). Of them, 13 participants underwent MRI scan of the brain using the ASL method.

The inclusion criteria for this study were as follows: (i) meeting the diagnostic criteria for major depressive disorder (MDD) according to the Diagnostic and Statistical Manual of Mental Disorders, 5th edition (DSM‐5)^21^; (ii) 14 or more points on the HAM‐D_17_ for depression severity; (iii) between 20 and 70 years of age at the time of study entry; (iv) a recent depressive episode of less than 3 years; and (v) not responding to sufficient dose and duration of pharmacotherapy with one or more antidepressants during the current depressive episode (or being unable to take a sufficient dose of pharmacotherapy due to intolerance to antidepressants). The exclusion criteria were as follows: (i) treatment history of electroconvulsive therapy, (ii) bipolar disorder, (iii) history of epilepsy or convulsive disorder, (iv) neurological diseases, (v) drug or alcohol dependence, (vi) serious physical diseases, and/or (vii) strong suicidal ideation. Psychotropic medications, including antidepressants, were fixed during the rTMS study period.

### 2.2 rTMS protocol and stimulation parameters

The Magstim 200 Square (The Magstim Company Ltd) stimulator was used for rTMS treatment. Stimulation parameters were based on the research protocols of O'Reardon et al.^22^ and George et al.,^23^ with a stimulation intensity of 100%–120% resting motor threshold (RMT). The stimulation frequency was set at 10 Hz, 75 trains per session, 4 seconds per train, and the stimulation interval was 26 seconds. A total of 3000 pulses were given to the left DLPFC as the target region. The targeted DLPFC stimulation site was 5 cm anterior to the parasagittal line from the target in the left primary motor cortex, which signaled the right abductor pollicis brevis muscle to contract.^24^, ^25^ The rTMS was performed one session per day on weekdays, five days per week for four weeks, for a total of 20 sessions (60 000 pulses).

### 2.3 Primary outcomes

The HAM‐D_17_ was used to assess depression severity^20^ and clinical assessments were performed before the start (week 0: baseline), week 2 (intermediate), and week 4 (final time) of the rTMS treatment. MRI scans were performed using a pre‐post design at weeks 0 and 4, although a range of 1 to 5 days was considered as an allowance period for the timing before and after treatment. All patients underwent MRI examination on a 3‐T imager (Signa HDxT3.0, General Electric Healthcare, Milwaukee, WI, USA) with an 8‐channel head array coil. The rCBF was assessed by the ASL, which is an MRI technique: magnetically labeling the spins of water molecules with a radiofrequency pulse and using it as an endogenous tracer in the cervical arteries.^26^, ^27^ A 3D spin‐echo, a pseudo‐continuous ASL (pCASL) sequence, was used for the ASL,^28^, ^29^ and the parameters were as follows: field of view, 24 cm; slice thickness, 4.0 mm; 32–38 slices; matrix (spiral points × arms) 512 × 8 × 36; number of excitations = three; and post‐labeling delay (PLD), 1.5 and 2.5 seconds. rCBF was calculated using the fine stereotactic region of interest template (FineSRT) program (PDRadiopharma Inc., Tokyo, Japan), which automatically analyzes the region of interest by segmenting into a total of 52 regions: 46 regions corresponding to each individual gyri and six major Broadman areas.^30^, ^31^ In this study, we defined the superior frontal, medial prefrontal, middle frontal, and inferior frontal as the DLPFC and the anterior cingulate, subcallosal, orbital, and rectal as the VMPFC, following previous studies^16^, ^19^ .

### 2.4 Statistical analysis

Longitudinal comparisons were conducted using one‐way repeated measures analysis of variance (ANOVA) with respect to the HAM‐D_17_ scores at three time points: baseline (week 0), mid‐treatment (week 2), and final (week 4). For multiple comparisons, Bonferroni's correction was employed. In addition, we compared the baseline rCBF (week 0) with the final one (week 4) using the paired *t* test. Pearson's correlation coefficient was applied to analyze the correlations between the improvement in the HAM‐D_17_ score (i.e. HAM‐D_17_ score at week 0–score at week 4) and the rCBF baseline (week 0), and between the improvement and the changes in rCBF (rCBF at week 4–week 0). As we planned a hypothesis‐testing statistical analysis in this study, the significance level was set at *P* = 0.05. SPSS 28.0 (IBM SPSS Inc) was used for all statistical analyses.

## 3 RESULTS

**FIGURE 1:**
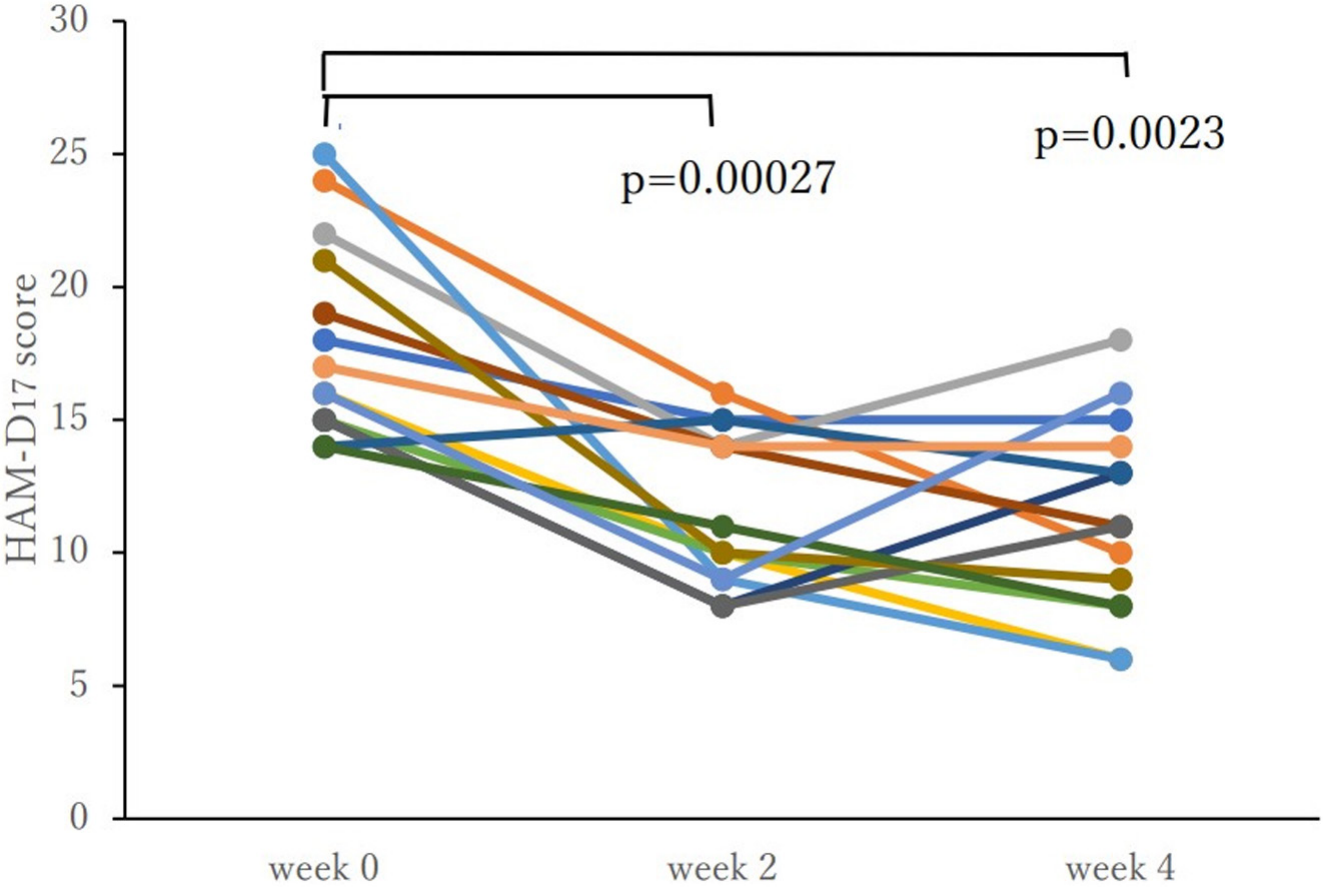
HAM‐D_17_ score changes for each participant. The HAM‐D_17_ score changes are shown in different colors for each case. In all but one case, the score improved from week 0 to week 4. Longitudinal analysis of the HAM‐D_17_ score showed a significant improvement at week 2 (*P* = 0.00027) and week 4 (*P* = 0.0023) compared with the baseline (week 0).

High‐frequency rTMS over 4 weeks resulted in a significant reduction in HAM‐D_17_ scores. Table 1 shows the HAM‐D_17_ scores at weeks 0, 2, and 4 of rTMS for the participants as well as the rCBF values in the DLPFC and VMPFC regions. We conducted an ANOVA for the HAM‐D_17_ score after starting rTMS, and the results showed a significant difference among the longitudinal changes [F (2, 24) = 18.663, *P* = 0.00001]. Multiple comparisons using Bonferroni's correction showed a significant reduction in depression severity at week 2 (*P* = 0.00027) and week 4 (*P* = 0.0023) when compared with week 0 (week 0: 17.9 ± 3.9; week 2: 11.4 ± 2.8; week 4: 11.0 ± 3.7), while no significant change was observed between weeks 2 and 4 in the HAM‐D_17_ scores. The HAM‐D_17_ score for each case individually showed an improvement at week 4 compared with week 0 in all but one (Figure 1). There were no significant changes in rCBF at each region in the pre‐post design.

**TABLE 1:** HAM‐D_17_ score and measurement parameters of rCBF at each measurement time point

	Brain region	PLD (second)	Measurement time point		
			Week 0	Week 2	Week 4
HAM‐D17 score(n = 14)			17.9 ± 3.9	11.4 ± 2.8*	11.0 ± 3.7*
rCBF (n = 13)	Right DLPFC	1.5	54.7 ± 10.4	N/A	53.5 ± 14.8
		2.5	56.0 ± 9.3	N/A	53.6 ± 11.0
	Left DLPFC	1.5	55.7 ± 11.8	N/A	54.2 ± 15.4
		2.5	55.9 ± 9.7	N/A	54.3 ± 11.8
	Right VMPFC	1.5	57.6 ± 8.5	N/A	56.3 ± 13.5
		2.5	60.4 ± 9.1	N/A	57.5 ± 10.2
	Left VMPFC	1.5	56.7 ± 8.3	N/A	55.8 ± 12.6
		2.5	57.9 ± 7.2	N/A	56.7 ± 9.3

*Note*: Mean ± SD; *P < 0.01: Comparison with week 0 using multiple comparisons using Bonferroni's correction for each week.

Abbreviations: DLPFC: dorsolateral prefrontal cortex; HAM‐D_17_: 17‐item Hamilton Depression Rating Scale; N/A: not applicable; PLD: post labeling delay; rCBF: regional cerebral blood flow; VMPFC: ventral medial prefrontal cortex.

**FIGURE 2:**
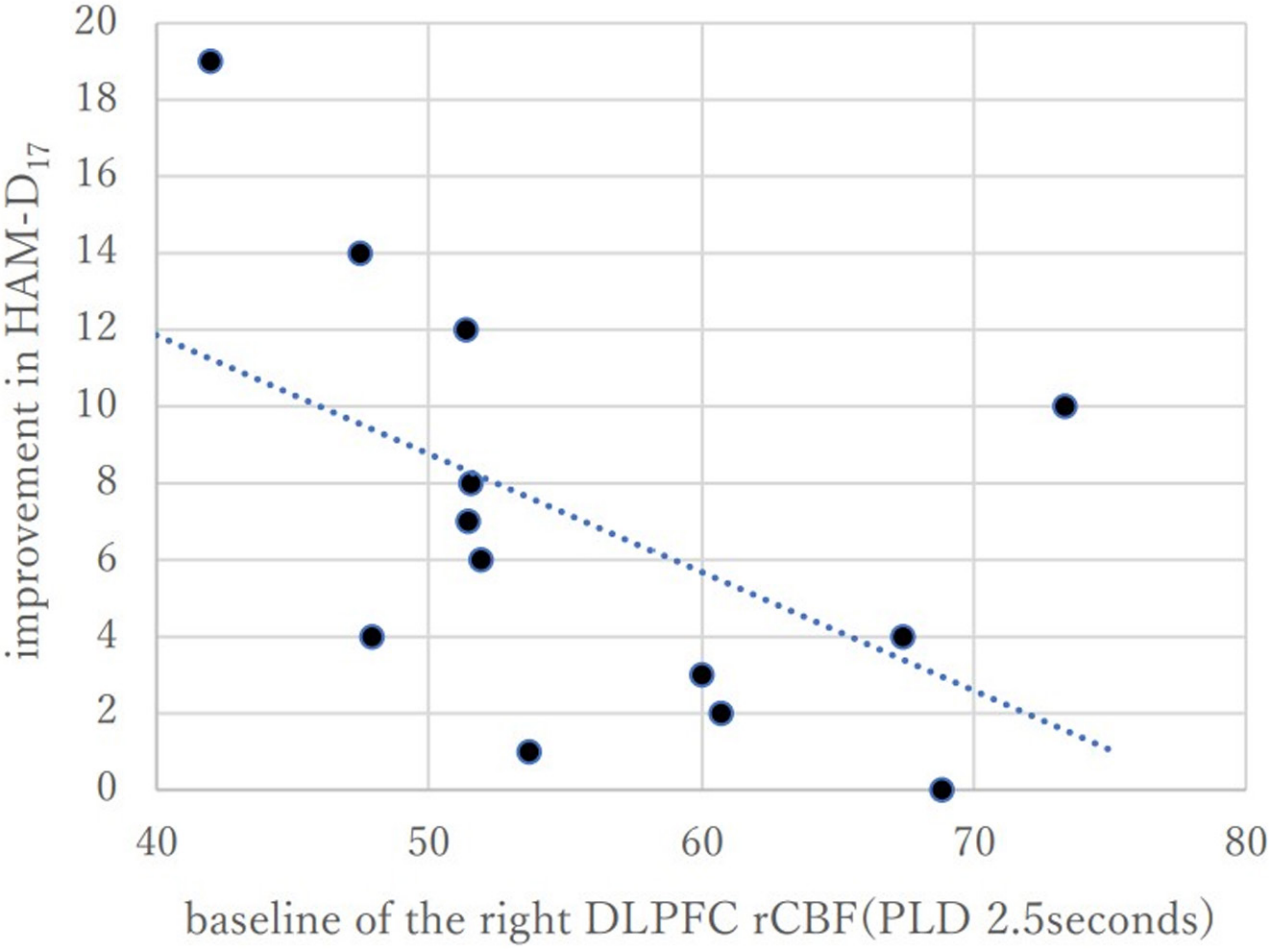
Scatter plot showing the relationship between the baseline rCBF (PLD 2.5 seconds) in the right DLPFC and the improvement in HAM‐D_17_ score. There was a significantly negative correlation between the baseline right DLPFC‐rCBF (PLD 2.5 seconds) and the improvement in the HAM‐D_17_ score (*r* = −0.559, *P* = 0.047, N = 13).

Table 2 shows results of the correlation analyses between the improvement in the HAM‐D_17_ score and the baseline rCBF, and between the improvement and change in rCBF. In the right DLPFC (PLD 2.5 seconds), there was a significant negative correlation between the baseline rCBF and the improvement in HAM‐D_17_ score (*r* = −0.559, *P* = 0.047, N = 13; Table 2, Figure 2). However, no significant findings were obtained in other correlation analyses.

**TABLE 2:** Correlation analyses between the improvement in HAM‐D_17_ score and the baseline rCBF as well as the change in rCBF

	rCBF							
Brain region	DLPFC							
	Right				Left			
	baseline		changes in rCBF^a^		baseline		changes in rCBF^a^	
PLD (seconds)	1.5	2.5	1.5	2.5	1.5	2.5	1.5	2.5
Improvement in HAM‐D_17_score^b^	−0.469	−0.559*	−0.004	0.247	−0.372	−0.466	−0.008	0.321
Brain region	VMPFC							
	Right				Left			
	baseline		changes in rCBF^a^		baseline		changes in rCBF^a^	
PLD (seconds)	1.5	2.5	1.5	2.5	1.5	2.5	1.5	2.5
Improvement in HAM‐D_17_score^b^	−0.484	−0.451	−0.097	0.417	−0.401	−0.465	−0.104	0.128

*P < 0.05.

^a^ rCBF at week 4–week 0.

^b^ HAM‐D_17_ score at week 0–score at week 4.

## 4 DISCUSSION

In this clinical study, rTMS for patients with MDD reflected a reduction in the HAM‐D_17_ score, indicating a significant improvement in depressive symptoms in the first half of the treatment period (within 2 weeks). This result indicates that 10 Hz‐rTMS to the left DLPFC is effective for depression, which is consistent with the meta‐analysis results of previous studies.^18^

To the best of our knowledge, this is the first study in Japan to assess rCBF by applying the MRI ASL method in the context of rTMS studies for depression and to examine its relationship with treatment response. We found that the improvement in the HAM‐D_17_ score with high‐frequency rTMS correlated with the rCBF value in the right DLPFC (PLD 2.5 seconds) at baseline before the commencement of rTMS. The lower the rCBF in the right DLPFC region at baseline, the greater the improvement in HAM‐D_17_ score with rTMS. Therefore, rCBF in the right DLPFC may be a clinical indicator for predicting treatment response to rTMS in patients with MDD. Herrington et al.^32^ conducted a fMRI study in which the participants identified the color of the unpleasant words printed and the depression group showed more right‐lateralized activation than controls in a neighboring DLPFC area. Nitschke et al.^33^ also observed an association between right prefrontal activity during the sad narrative and memory performance in their electroencephalogram study. Thus, depression‐related activity in the right DLPFC is considered to be associated with negative affect. Based on these previous findings, decreased rCBF in the right DLPFC, reflecting brain activity, may be a subtype of depression in which negative affect is low. That may explain generally low resistance to treatment and relatively high response to rTMS observed in our study.

Kito et al.^19^ conducted high‐frequency rTMS in 24 Japanese patients with treatment‐resistant depression and evaluated rCBF using single‐photon emission computed tomography (SPECT) before and after treatment. They observed no significant correlation between the rCBF of the DLPFC before rTMS and the rate of decrease in the HAM‐D_17_ score (= the improvement in HAM‐D_17_ score/score at week 0). However, when evaluated by the ratio of rCBF of DLPFC to rCBF of VMPFC before rTMS (DLPFC/VMPFC), a significant negative correlation was found between the ratio and rate of decrease in the HAM‐D_17_ score, regardless of the laterality of the DLPFC/VMPFC ratio. Therefore, the smaller the DLPFC/VMPFC ratio, the better the treatment response.^19^ In the present study, we found a significant negative correlation between the baseline rCBF of the right DLPFC and the improvement in HAM‐D_17_ score, while no significant clinical correlation was observed with respect to the DLPFC/VMPFC ratio (data not shown). Furthermore, in the present study, there were no significant changes in rCBF in the DLPFC and VMPFC regions throughout the course of rTMS, and no significant correlation was found between the improvement in HAM‐D_17_ score and the change in rCBF in the DLPFC or the VMPFC. In contrast, Kito et al.^3^ observed that rCBF in the left DLPFC significantly increased throughout the course of rTMS in 12 patients with treatment‐resistant depression. Furthermore, there was a significant correlation between the change in HAM‐D_17_ scores and that in rCBF of the left DLPFC in the responder group in their study.^3^

There are thus some discrepancies between the present and previous results.^3^, ^19^ One of the reasons may be the difference in inclusion criteria of patients with depression among the studies. Hence, differences in the degree of medication resistance and the mean period of depression may naturally lead to some differences in treatment response to rTMS and changes in rCBF. Another reason may be the difference in the measuring method of rCBF; in the present study, we used the more non‐invasive MRI ASL method, whereas PET and SPECT have been the most common measurement modalities for rCBF in previous studies.^3^, ^19^ Since it has been reported that there is a significant correlation between the rCBF measured by ASL and that measured by PET,^34^ the effect of the methodological difference may be negligible. However, the MRI ASL method remains susceptible to artifacts.^26^, ^35^ Specifically, the PLD setting has a significant impact on the accuracy of signal measurement. If there is stenosis in the major blood vessels, the time to reach the region of interest may increase, and the accuracy may decrease with a single PLD measurement.^26^, ^35^ Therefore, in this study, we measured with a PLD of 1.5 and 2.5 seconds. It is possible that our settings of the ASL imaging parameters might not have necessarily been optimized. It may be necessary to accumulate knowledge on the appropriate PLD setting method to improve the accuracy of rCBF measurement using the ASL method in a future study.

There are several limitations in this study. First, the sample size was small, which is subject to type II errors. Second, this was an open‐label study and did not include a placebo control group with sham stimulation. Therefore, it may be necessary to conduct more rigorous ASL measurements in randomized controlled trials (RCTs) with larger sample sizes. Third, MRI neuronavigation was not used for rTMS in this study; therefore, the identification of the stimulation sites in the left DLPFC may not be optimized.^36^

## 5 CONCLUSION

In the present study, high‐frequency rTMS was performed in patients with MDD, and we obtained supportive evidence for its antidepressant effects consistent with the results in previous rTMS studies.^18^ Decreased rCBF in the right DLPFC (i.e., hypoactivity of the right DLPFC) before the initiation of rTMS may indicate that the therapeutic effect of rTMS is more likely to be achieved. Considering that hyperactivity of the right DLPFC region has been assumed in depression, this finding may be a clinical indicator for predicting the therapeutic effect of rTMS in patients with depression. Future studies with larger sample sizes and RCT design with a placebo group are needed to confirm the reproducibility of our results.

## AUTHOR CONTRIBUTIONS

HI and TM contributed to the conception, design, and statistical data analysis of the study, performed the experiments, and wrote the first draft of the manuscript. YN revised the manuscript critically for intellectual content and approved the submitted version. HO, TK, and KS contributed to the acquisition of the data and the conception and design of the study. EI and NH contributed to the conception and design of the study. HK revised the manuscript critically for intellectual content and approved the submitted version.

## CONFLICTS OF INTEREST

The authors declare no conflicts of interest regarding this paper.

## APPROVAL OF THE RESEARCH PROTOCOL BY AN INSTITUTIONAL REVIEW BOARD

This study was reviewed and approved by the Institutional Review Board of Teikyo University School of Medicine (Teirin 13–026‐5 and 19–060).

## INFORMED CONSENT

The study content was explained to the participants verbally and in writing, and all participants gave written consent.

## Data Availability

The data cannot be made publicly available as data sharing was not included in the consent form.
